# F-Actin Dysplasia Involved in Organ of Corti Deformity in *Gjb2* Knockdown Mouse Model

**DOI:** 10.3389/fnmol.2021.808553

**Published:** 2022-03-07

**Authors:** Xiao-zhou Liu, Yuan Jin, Sen Chen, Kai Xu, Le Xie, Yue Qiu, Xiao-hui Wang, Yu Sun, Wei-jia Kong

**Affiliations:** ^1^Department of Otorhinolaryngology, Union Hospital, Tongji Medical College, Huazhong University of Science and Technology, Wuhan, China; ^2^Department of Otolaryngology, Head and Neck Surgery, The Second Affiliated Hospital of Nanchang University, Nanchang, China; ^3^Institute of Otorhinolaryngology, Tongji Medical College, Huazhong University of Science and Technology, Wuhan, China

**Keywords:** connexin26, GJB2, hearing loss, cochlear development, f-actin

## Abstract

Mutations in the *GJB2* gene encoding connexin26 (Cx26) protein are one of the most common causes of hereditary deafness. Previous studies have found that different Cx26-null mouse models have severe hearing loss and deformity of the organ of Corti (OC) as well as a reduction in microtubules in pillar cells (PCs). To explore the underlying mechanism of OC deformity caused by Cx26 downregulation further, we established Cx26 knockdown (KD) mouse models at postnatal days (P)0 and P8. The actin filaments contained in the pillar cells of mice in the P0 KD group were reduced by 54.85% and vinculin was increased by 22%, while the outer hair cells (OHCs) showed normal F-actin content. In the P8 KD group, PCs and OHCs of mice also showed almost normal F-actin content. The G-actin/F-actin ratio increased by 38% in the P0 KD group. No significant change was found in the mRNA or protein expression level of G-actin or the cadherin–catenin core complex in the P0 KD group at P6. Moreover, immunofluorescence showed that the intensity of LRRK2 was reduced by 97% in the P0 KD group at P6. Our results indicate that Cx26 is involved in the maturation of the cytoskeleton during the development of the OC at the early postnatal stage. The polymerization of G-actin into F-actin is prevented in Cx26 KD mice.

## Introduction

Hearing loss is the most common congenital sensory disorder (Chan and Chang, 2014). Approximately one–two out of every 1,000 newborns have hearing loss (Sun et al., 2009). The genes encoding connexins are the most common genetic causes of congenital hereditary deafness (Martínez et al., 2009). Six connexins form a hemichannel, and two hemichannels from a pair of adjacent cells form a gap junction (Harris, 2001). Gap junctions are channels for material exchange and communication between cells, which allow ions, microRNAs, second messengers, and small molecules ≤1.5 kDa to pass through (Zhu et al., 2015). In the inner ear of mammals, connexin26 (Cx26) encoded by the *GJB2* gene is one of the most widely-distributed gap junction proteins (Ahmad et al., 2003). Mutations in the *GJB2* gene are the most common cause of human autosomal hereditary hearing loss. However, the mechanism is still not completely clear.

Mutations in *GJB2* cause variable levels of congenital hearing loss (Rabionet et al., 2000). Due to the difficulty in obtaining human pathological specimens, the mechanism of *GJB2* gene mutation-induced deafness is mainly studied using the *GJB2* gene knock-down mouse model (Qu et al., 2012; Chang et al., 2015; Chen et al., 2018a; Xie et al., 2019). We and others have found the failure of the tunnel of Corti (TC) to open and the disappearance of Nuel’s space (Ns) in *Gjb2* KD mice (Chen et al., 2018b). Abnormal development of the cytoskeleton in PCs seems to be the main reason for the deformity of OC (Chen et al., 2018b). Actin filaments and microtubules jointly form a cytoskeletal network. Previous research has shown that the stereocilia structure of hair cellis formed from actin filaments which are composed of a mixture of β-actin and γ-actin, and mutations in the gene encoding actin can cause hereditary deafness (Patrinostro et al., 2018). However, few researchers have focused on the actin filaments of the inner ear supporting cells.

Mice with Cx26 KD before P4 suffer from severely impaired hearing, with deformity of the OC and reduction of microtubules in the pillar cells (Wang et al., 2009). In addition, Deiter’s cells (DCs) are unable to develop into finger-like structures (Chen et al., 2018b). However, the mice with knockdown of Cx26 after P6 show normal hearing and the OC displays normal architecture. Therefore developmental abnormality of OC might be a better explanation for the hearing loss caused by Cx26 KD at the early postnatal stage. To explore the underlying mechanism of OC deformity caused by Cx26 downregulation further, we established mice that knockdown Cx26 at P0 and P8. We analyzed the composition of actin filaments such as β-actin and γ-actin, main ingredients of the cadherin–catenin core complex and molecules that are involved in the regulatory mechanism of microfilament assembly in Cx26 KD mice. We found a decrease in F-actin with an increase of vinculin and a reduction of LRRK2 in P0 knockdown mice. Abnormal development of microfilaments may be involved in the mechanism of deafness caused by Cx26 KD. In addition, reduction of LRRK2 may be the cause of the reduction of F-actin in the P0 KD group.

## Materials and Methods

### Mouse Models

Cx26loxP/loxP mice and Rosa26CreER mice were provided by Prof. Xi Lin at Emory University. Tamoxifen-inducible Cx26loxP/loxP; Rosa26CreER mice were generated by crossbreeding of the Cx26loxP/loxP mice with Rosa26CreER mice. Mouse genotyping was performed by PCR amplification of tail genomic DNA. The methods of breeding and genotyping the mice are detailed in our previous article (Chen et al., 2018b). The genotyping primers were as follows:

Cx26 (F): 5’-ACAGAAATGTGTTGGTGATGG-3’,

Cx26 (R): 5’-CTTTCCAATGCTGGTGGAGTG-3’,

Rosa26Cre (F): 5’-AGCTAAACATGC

TTCATCGTCGGTC-3’,

Rosa26Cre (R): 5’-TATC

CAGGTTACGGATATAGTTCA TG-3’,

In this experiment, to observe the expression pattern of Cx26, a smaller dose of TMX (total dose 1.5  mg/10  g per day for 2 days) was administered to Cx26loxP/loxP; Rosa26CreER mice at P0/P1(P0 KD Group) and P8/P9 (P8 KD Group), and cochlear Cx26 was partly and randomly knocked out in different types of cells of this group (the randomly Cx26-null group).

All mice were raised in a sterile constant temperature environment at the specific-pathogen-free Experimental Animal Centre of Huazhong University of Science and Technology. All experimental work passed the ethical review of the animal experiments and was conducted in accordance with the policies of the Committee on Animal Research of Tongji Medical College, Huazhong University of Science and Technology.

### Auditory Brainstem Response

Auditory brainstem response (ABR) was examined at P20. Mice were treated with combined anesthesia by intraperitoneal injection with ketamine (120 mg/kg) and chlorpromazine (20 mg/kg). Anaesthetised mice were then placed on an electric blanket to maintain body temperature at 37–38°C. Thresholds evoked by tone bursts of 8, 16, 24, 32, and 40 kHz were generated and responses were recorded using a Tucker-Davis Technologies system (RZ6, Tucker-Davis Tech., Alachua, FL, USA). If any mice suffered from severe hearing loss, the ABR test in the intensity range of 50–90 dB SPL was used. The lowest sound level that could be recognized was considered to be the auditory threshold.

### Transmission Electron Microscopy (TEM)

This process has been described in detail in previous studies (Chen et al., 2018b). After the animals were euthanized, a hole was excavated using tweezers at the apex cochleae and then the cochleae were soaked in a mixture of 2% paraformaldehyde and 2.5% glutaraldehyde in 0.1 M phosphate-buffered saline (PBS). The cochleae were decalcified for 48 h in 10% disodium EDTA (pH 7.2). Next, the sample was soaked in 1% osmium tetroxide. After dehydration, the sample was embedded in resin and sectioned, and the ultrathin sections were stained with uranyl acetate and lead citrate. Electron microscopy examination (FEI Tecnai G2 20 TWIN; Hillsboro, OR, USA) was used to observe the ultrathin sections. The OC was magnified ×420 and images were captured for examination.

### Western Blot

After the animals were euthanized, the membranous labyrinths of the cochleae were dissected carefully in ice-cold 1% PBS. Two membranous labyrinthine tissues from each mouse’s left and right cochleae were combined to generate one sample. During the dissection, the tube containing the tissue was placed on ice. For cochlear explants, each of two basement membranes was considered to constitute a sample. Membranous labyrinths and cochlear explants were lysed with cold RIPA lysis buffer containing 1% PMSF. A BCA protein quantification kit (Beyotime Biotechnology, Jiangsu, China; P0010) was used to measure the protein concentration. Equal amounts of protein (5 μg) were separated by gel electrophoresis on a 12% sodium dodecyl sulfate (SDS) polyacrylamide gel, then electroblotted onto a polyvinylidene difluoride (PVDF) membrane. Proteins were then detected using the following antibodies: anti-GAPDH rabbit monoclonal antibody (ANT324; Antgene, Biotechnology Company Ltd, Wuhan, China); anti-β-actin rabbit polyclonal antibody (ANT322, Antgene); anti-gamma-actin rabbit polyclonal antibody (11227-1-AP; Proteintech, Rosemont, IL, USA); anti-Cx26 rabbit polyclonal antibody (71-0500; Invitrogen, Carlsbad, CA, USA); anti-H3 histone rabbit antibody (A2348; ABclonal, Woburn, MA, USA); anti-α-catenin rabbit antibody (A19004, ABclonal); anti-LRRK2 rabbit polyclonal antibody (A17253, ABclonal); anti-RHOA rabbit antibody (A17253, ABclonal); anti-Arp2 rabbit antibody (A5734, ABclonal); anti-Arp3 rabbit antibody (A1046, ABclonal); anti-Vinculin rabbit antibody (A14193, ABclonal); anti-β-catenin rabbit antibody (YT0676, Immunoway, Plano, TX, USA); anti-p120-ctn rabbit antibody (12180-1-AP, Proteintech); anti-α-catenin rabbit antibody (YT0676, Immunoway); the dilution ratio of all solutions containing primary antibodies was 1:1,000. Briefly, GAPDH and histone H3 were used as the reference proteins in line with the manufacturer’s instructions. A SuperSignal West Dura chemiluminescent substrate kit was used to detect the complexes according to the manufacturer’s instructions. The western blots were semi-quantified using Image J (NIH, Bethesda, MD, USA) to measure the intensities of the bands.

### RNA Preparation and Real-Time Quantitative Polymerase Chain Reaction (RT-qPCR)

The procedure used for RT-qPCR was as described in a previous article (Xu et al., 2021). RT-qPCR was performed to determine the transcriptional expression level of the following genes: *Gjb2*, *Acta1*, *Acta2*, *Actb*, *Actg1*, *Actg2*, *Actc1*, *Cdh1* (E-cadherin), *Ctnna1* (α-catenin), *Ctnnb1* (β-catenin), and *Ctnnd1* (p120-ctn). After the animals were euthanized, the membranous labyrinths of the cochleae were dissected carefully on ice. The membranous labyrinthine tissues from each mouse’s left and right cochleae were used to generate one sample. There were six biological replicates for each experimental condition. Total RNA was extracted from the collected tissues using an RNAprep Pure Tissue Kit (Tiangen Biotech Co. Ltd., Beijing, China) and was reverse transcribed using a PrimeScript RT Reagent Kit with gDNA eraser (Takara Bio Inc., Shiga, Japan). Real-time PCR was performed in a LightCycler 480 instrument (Roche, Basel, Switzerland). Analysis of relative gene expression data between sample groups was performed according to the standard 2–ΔΔCP method. The following primers were used for RT-qPCR:

Gjb2 (F): 5’- CTCGGGGGTGTCAACAAACA-3’,

Gjb2 (R): 5’- CACGAGGATCATGATGCGGA-3’,

Acta1 (F): 5’- GAGCGTGGCTATTCCTTCGT-3’,

Acta1 (R): 5’- GAAACGCTCATTGCCGATGG-3’,

Acta2 (F): 5’- GTACCACCATGTACCCAGGC-3’,

Acta2 (R): 5’- GCTGGAAGGTAGACAGCGAA-3’,

Actg2 (F): 5’-ACAGAAATGTGTTGGTGATGG-3’,

Actg2 (R): 5’- TCTTCTGGTGCTACTCGAAGC-3’,

Actg1 (F): 5’- GAGCAAGAAATGGCTACTGCTG-3’,

Actg1 (R): 5’- AGCAATGCCTGGGTACATGG-3’,

Actb (F): 5’- GCGGGCGACGATGCT-3’,

Actb (R): 5’- GCCACAGGATTCCATACCCA-3’,

Actc1 (F): 5’- AAACTGTGTTACGTCGCCCT-3’,

Actc1 (R): 5’- GGGCCTGCCTCATCATACTC-3’,

Gapdh (F): 5’- GAAGGTCGGTGTGAACGGAT-3’,

Gapdh (R): 5’- CTCGCTCCTGGAAGATGGTG-3’,

Ctnnb1 (F): 5’- ATGGAGCCGGACAGAAAAGC-3’,

Ctnnb1 (R): 5’- TGGGAGGTGTCAACATCTTCTT-3’,

Ctnna1 (F): 5’- GTCCACGCAGGCAACATAAAC-3’,

Ctnna1 (R): 5’- CTGTGTAACAAGAGGCTCCAAC -3’,

Ctnnd1 (F): 5’- GTGGAAACCTACACCGAGGAG -3’,

Ctnnd1 (R): 5’-CTTTCCAATGCTGGTGGAGTG-3’,

Cdh1 (F): 5’-AACCCAAGCACGTATCAGGG-3’,

Cdh1 (R): 5’-ACTGCTGGTCAGGATCGTTG-3’,

### Immunofluorescence Staining

After the animals were euthanized, the cochleae were carefully dissected from the temporal bones and fixed in 4% paraformaldehyde in 0.01 M PBS at room temperature for 2 h. The apical basement membranes were dissected in 0.01 M PBS. The tissues were incubated in a blocking solution (10% donkey serum with 0.1% Triton X-100) for 1 h at room temperature, and then placed in antibody solution (diluted in 1% BSA in PBST) and incubated overnight at 4°C. The next day, the tissue was placed on a shaker at room temperature for 1 h, then washed four times with 0.01 M PBS for 10 min each time. Samples were then incubated for 2 h at room temperature in 0.01 M PBS containing the secondary antibody. After repeating the washing step, DAPI (4’,6-diamidino-2-phenylindole dihydrochloride) and phalloidin were dripped onto the tissue before covering with a coverslip and incubating for 2 h in the dark at room temperature. The following antibodies and reagents were used for immunofluorescence staining in this study: anti-Cx26 mouse polyclonal antibody (1:300, 131800, Invitrogen), LRRK2 (1:200, A0859, ABclonal), Alexa Fluor 647 donkey anti-rabbit IgG (1:200, ANT032S, Antgene), or Alexa Fluor 568 goat anti-Mouse IgG (1:200, RS3508, Immunoway). DAPI (C1005; Beyotime Biotechnology) and phalloidin (40736ES75; Yeasen, Shanghai, China) were used for nuclear and F-actin staining, respectively. Images were obtained with a laser scanning confocal microscope (Nikon, Tokyo, Japan).

### G-Actin/F-Actin Ratio Measurement

A G-Actin/F-actin *in vivo* Assay Biochem Kit (Cytoskeleton Inc., Denver, CO, USA; BK037) was used to evaluate the G-Actin/F-actin ratio of the cochlea. G-actin and F-actin proteins were separated according to the instructions. In brief, the membranous labyrinths of the cochleae were dissected in 0.01 M PBS. Tissues were frozen in liquid nitrogen and ground into a powder. LAS2 buffer (1 ml per 0.1 g of tissue) was added to the sample, then F-actin Enhancing Solution was added to the sample at a volume ratio of 1:100. The tissue was lysed in the mixed solution for 10 min at 37°C, then the lysate was placed in a centrifuge and centrifuged at 2,000 rpm for 5 min to remove impurities after lysis. The supernatants were then pipetted into clearly-labeled ultracentrifuge tubes, and centrifuged at 100,000× *g*, 37°C for 1 h. The supernatant, which contained G-actin, was separated and the F-actin depolymerization buffer was added to the precipitate. The sample was placed on ice for 1 h, inverting the sample every 15 min, to obtain the G-actin solution produced by the depolymerization of F-actin. Finally, 5× SDS sample buffer was added to each tube, and western blotting was used to evaluate the G-actin/F-actin ratio.

### Data Analysis

All data are presented as means ± SE and plotted using GraphPad Prism (Version 8.0.1, GraphPad Software Inc., La Jolla, CA, USA). Student’s t-tests or correlation analyses were performed in SPSS software (Version 19, IBM SPSS Statistics, IBM Corp., Armonk, NY, USA), and *p* < 0.05 was considered to be statistically significant.

## Results

### Hearing Loss and Deformity of the Organ of Corti Were Observed in Mice Only When *Gjb2* Was Knockdown at the Early Postnatal Stage

**Figure 1:**
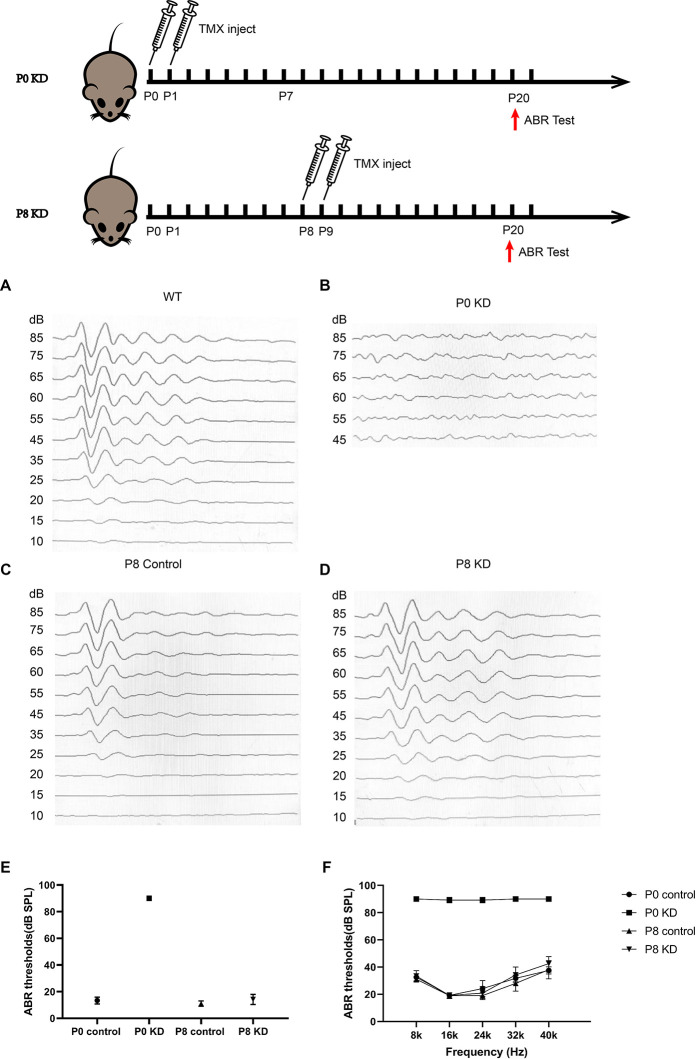
Mice that Cx26 were knockdown at P0 have severely impaired hearing. Auditory thresholds (*n* = 6 in each group) evoked by click **(A–E)** or tone-bursts **(F)** were measured in the P0 control, P0 KD, P8 control,and P8 KD groups. All ABR tests were performed at P20.

**Figure 2:**
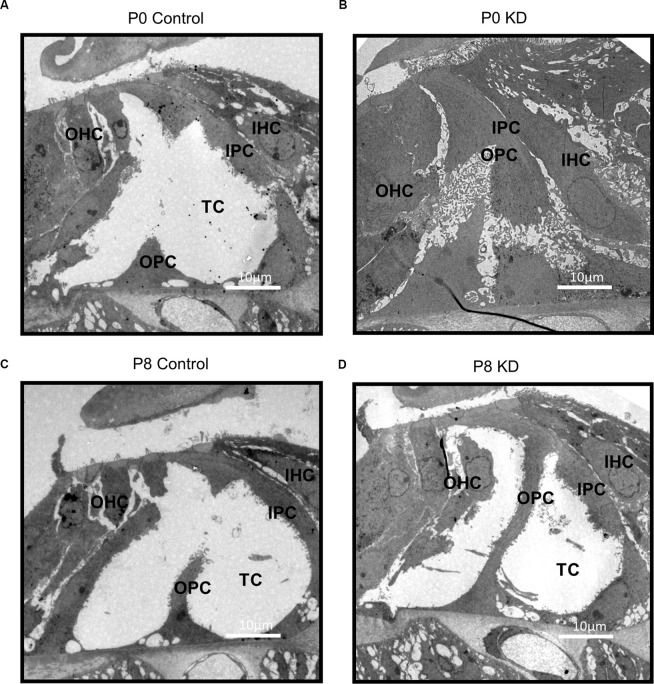
Deformity of OC was only observed in P0 KD Group. A full view of the OC sections obtained from the P0 control **(A)**, P0 KD groups **(B)**, P8 control **(C)**, and P8 KD **(D)**. Scale bar: ~10 μm **(A–D)**. DC, Deiter’s cells; IHC, inner hair cells; OHC, outer hair cells; IPC, inner pillar cells; OPC, outer pillar cells; TC, tunnel of Corti; NS, Nuel’s space.

The hearing of mice gradually matures and stabilizes at P16–P18. During the development of the OC, the height of the OC increases and opens with the formation of the TC (Chen et al., 2014). In order to observe the effects of Cx26 knowdown at different stages on the hearing and the morphology of the OC in mice, auditory brainstem response (ABR) measurements and morphological observations were performed on P20 mice injected with tamoxifen (TMX) at P0 and P8. Consistent with previous reports, mice in the P0 KD group (*n* = 6) showed severe hearing loss at the frequency of detection. The ABR thresholds evoked by click stimulations in the P0 KD group (*n* = 6) remained above 90 dB sound pressure level (SPL) and thresholds evoked by tone-bursts across a range of frequencies (4 K, 8 K, 16 K, 32 K, 40 K) were up to at least 85 dB SPL (Figures 1A,B,E). Meanwhile, the ABR thresholds evoked by click stimulations in the P8 KD group (*n* = 6) were almost 15 ± 3.43 dB SPL (Figures 1C,D), and thresholds evoked by tone-bursts across a range of frequencies (4 K, 8 K, 16 K, 32 K, 40 K) were 33 ± 3.72, 19 ± 1.86, 19 ± 4.48, 32 ± 5.33, and 41 ± 4.78 dB SPL (Figure 1F). This shows that there was no significant difference in hearing between the Cx26 P8 KD group and the control group. Deformity of the OC was observed only in the P0 KD group by electron microscopy (Figures 2A–D).

### F-Actin Decreased by 54.85% in PCs of the P0 KD Group

**Figure 3:**
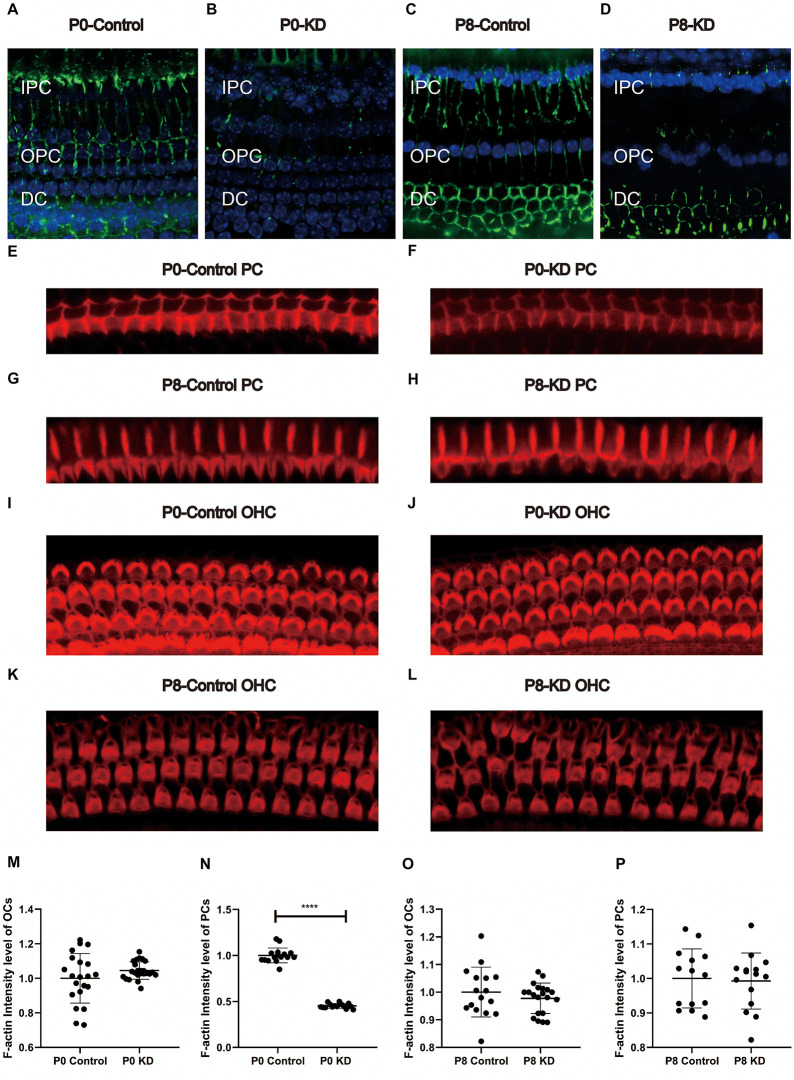
Immunofluorescence staining of Cx26 and F-actin at p6 in P0 KD and p8 KD group. Immunofluorescence staining of Cx26 of support cell in P0 control **(A)**, P0 KD groups **(B)**, P8 control **(C)**, and P8 KD **(D)**. Staining of microfilaments labeled at the top of the pillar cells with phalloidin in P0 control **(E)**, P0 KD groups **(F)**, P8 control **(G)**, and P8 KD **(H)**. Staining of microfilaments labeled at outer hair cells with phalloidin in P0 control **(I)**, P0 KD groups **(J)**, P8 control **(K)**, and P8 KD **(L)**. Statistics of F-actin fluorescence intensity of PCs or OCs (**M**–**P**; *n* = 3 in each group). All the staining were performed 7 days after injection of TMX. Scale bar: ~10 μm **(A–L)**. IPC, inner pillar cells; OPC, outer pillar cells; DC, Deiter’s cells. ****Significantly different from the control group (*P* < 0.0001).

Actin filaments, also known as F-actin, constitute an important part of the cytoskeleton. The actin cytoskeleton within a cell is necessary for the maintenance of cell shape, cell motility, and intracellular transport. A mixture of β-actin and γ-actin filaments formed stereocilia of hair cells and previously we have reported that the formation of the OC might be in association with the cytoskeleton in pillar cells (Dominguez and Holmes, 2011). The classic research method is to label the microfilament skeleton with fluorescently-labeled phalloidin (Prentki et al., 1979). No Cx26 expression was detected in inner pillar cells (IPCs) or outer pillar cells (OPCs) either in the P0 KD group or the P8 KD group (Figures 3A–D). The fluorescence intensity of phalloidin-labeled F-actin in the P0 KD group was reduced by 54.85% at P7 compared with the control group in both OPCs and IPCs (Figures 3E,F,N). In contrast, the P8 KD group showed no significant difference in the fluorescence intensity of phalloidin-labeled actin filaments at P15 (Figures 3G,H,P). However, no significant difference in F-actin staining of hair cells was observed between the P0 KD group and the P8 KD group (Figures 3I–M,O).

### The Ratio of G-Actin/F-Actin Increased in the P0 KD Group

F-actin is formed by polymerization of G-actin (Rottner et al., 2017). In order to perform these functions, the F-actin network must assemble and depolymerize at the right time and place. To verify whether the assembly process of F-actin is disturbed, we analyzed the G-actin/F-actin ratio in the P0 KD group. Compared with the control group, the ratio of G-actin/F-actin increased by 38% in the P0 KD group (Figures 5A,B). This result suggests that there may be interference in the process of assembly of G-actin into F-actin or depolymerization of F-actin into G-actin caused by Cx26 KD at the early postnatal stage.

**Figure 4:**
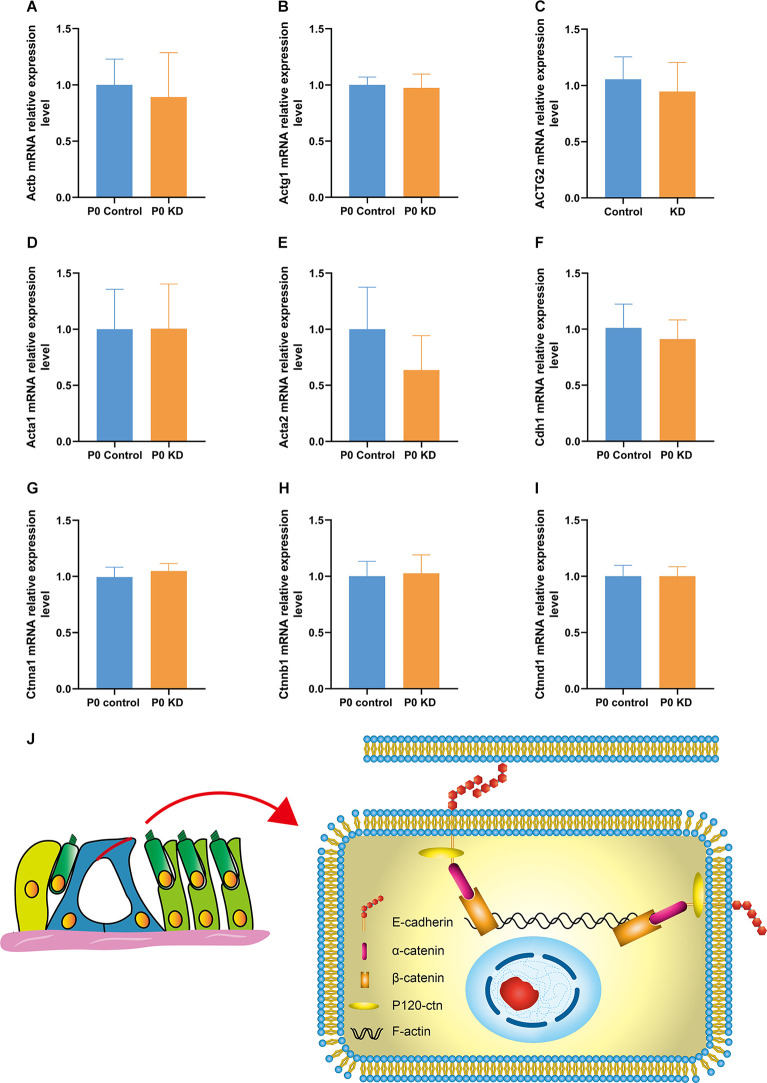
mRNA expression relative expression level of G-actin and the cadherin–catenin core complex in P0 KD group. mRNA expression level in P0 control and P0 KD groups **(A–I)**. The differences between the P0 KD group and the P0 control group were not significant (*P* > 0.05). Adhesive protein complexes at adherens junctions between IPCs and OPCs **(J)**.

**Figure 5:**
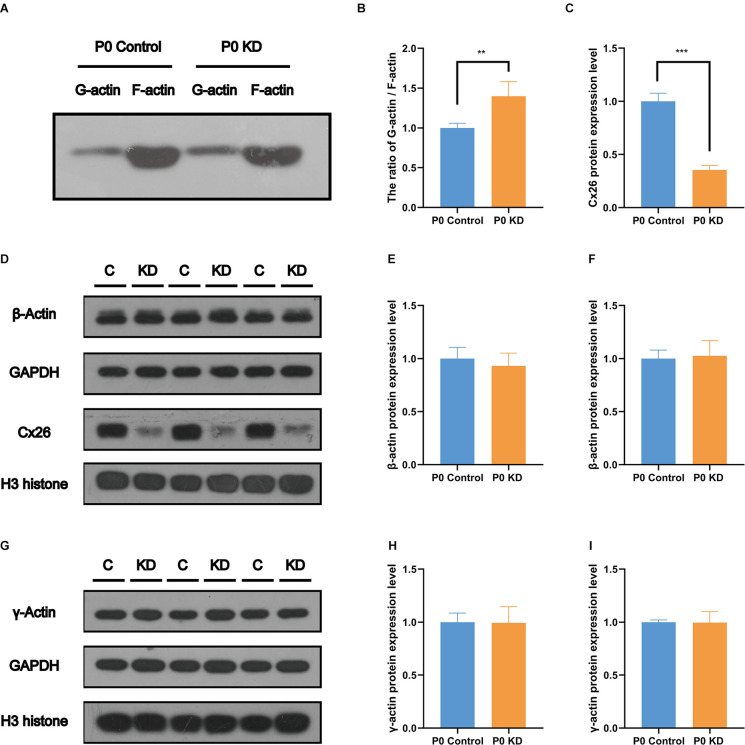
No change was found in the protein expression level of G-actin. Protein expression level of Cx26, β-actin **(A)**, and γ-actin **(E)** in P0 control and P0 KD groups. β-actin and γ-actin protein expression level in cochlear of Cx26 KD mouse models. The ratio of G-actin/F-actin in P0 control and P0 KD group **(B)** respectively. Western blotting F-actin and G-actin in Western blot results of protein expression level of Cx26, β-actin **(D)** and γ-actin **(G)** in P0 control and P0 KD groups. Camparison of relative Cx26 **(C)**, β-actin **(E,H)**, and γ-actin **(F,I)** protein expression in P0 control and P0 KD groups. GAPDH **(C,E,H)** and H3 **(F,I)** was used as the control, respectively. The differences of β-actin and γ-actin expression level between the P0 KD and the P0 control group were not significant (*P* > 0.05). ***Significant difference from the control group (*P* < 0.001). **Significant difference from the control group (*P* < 0.01).

### No Change Was Found in the mRNA or Protein Expression Level of G-Actin

The actin family includes α-actin, β-actin, and γ-actin (Dominguez and Holmes, 2011). In non-muscle tissues, F-actin is mainly composed of γ-actin and β-actin, which are encoded by *ACTG* and *ACTB* genes (Pollard and Cooper, 2009). In order to detect the effect of Cx26 KD on the transcription level of the gene encoding G-actin, we used Quantitative Real-time PCR (QPCR) to analyze the mRNA expression level of *Actg1*, *Actg2*, *Actb*, *Acta1*, *Acta2*, and *Actc1* in the P0 KD group at P7. Compared with the control group, there was no significant difference in the mRNA levels of* Actg1*, *Actg2*, *Acta1*, *Acta2*, or *Actb* (Figures 4A–E). However, we detected the mRNA transcript of Actc1 in only a few specimens. We speculate that actc1 might be expressed in spiral ganglion instead of the OC and samples may be accidentally contaminated with other tissues during anatomical sampling. Similarly, we measured the protein expression level of G-actin with H3 and Gpadh as internal reference proteins. The result showed that there were no significant differences between the protein expression levels of β-actin and γ-actin (Figures 5D–I).

### No Changes Were Found in the mRNA and Protein Expression Levels of the Cadherin–Catenin Core Complex

**Figure 6:**
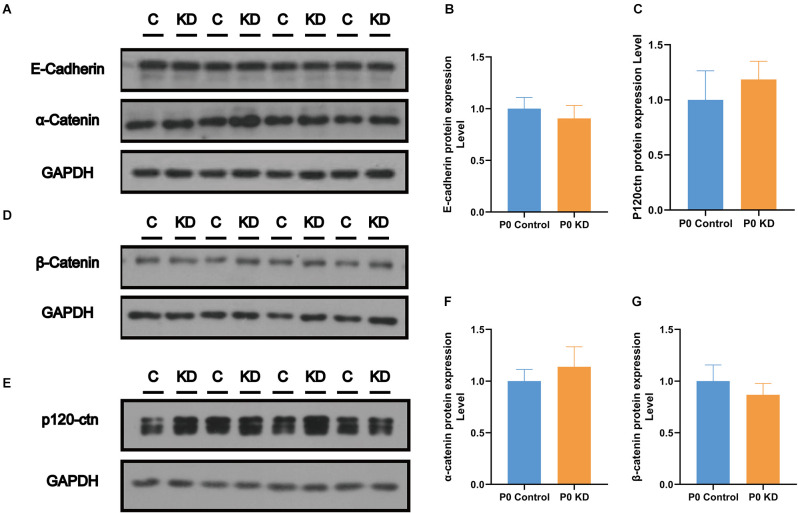
No change was found in the protein expression level of the cadherin–catenin core complex. Protein expression level of E-cadherin, α-catenin **(A)**, β- catenin **(D)**, and p120-ctn **(E)** in P0 control and P0 KD groups. Comparison of relative E-cadherin **(B)**, p120ctn **(C)**, α-catenin **(F)**, and β-catenin **(G)** protein expression in P0 control and P0 KD groups. GAPDH was used as the control.

The actin cytoskeleton is linked to the cadherin complex on the cell membrane (Figure 4J; Gloushankova et al., 2017). E-cadherin directly interacts with its two cytoplasmic binding partners p120ctn and β-catenin, and β-catenin interacts with α-catenin. Meanwhile, α-catenin interacts directly with F-actin, thereby linking E-cadherin to the junctional actin (Brieher and Yap, 2013). To investigate whether Cx26 KD affects the cadherin–catenin core complex, we analyzed mRNA and protein expression levels of the components of the cadherin complex such as E-cadherin, p120ctn, α-catenin, and β-catenin in the P0 KD group at P7. The QPCR results showed that, compared with the control group, there were no significant differences in the mRNA expression level of E-cadherin, α-catenin, β-catenin, or p120-catenin (Figures 4A–E). Western blot results show that in comparison with the corresponding control groups, Cx26 is significantly downregulated (Figures 5C,D), while there is no significant difference in the protein expression level of E-cadherin, p120-cadherin, α-catenin or β-catenin (Figures 6A–G).

### Vinculin Increased in PCs of the P0 KD Group

**Figure 7:**
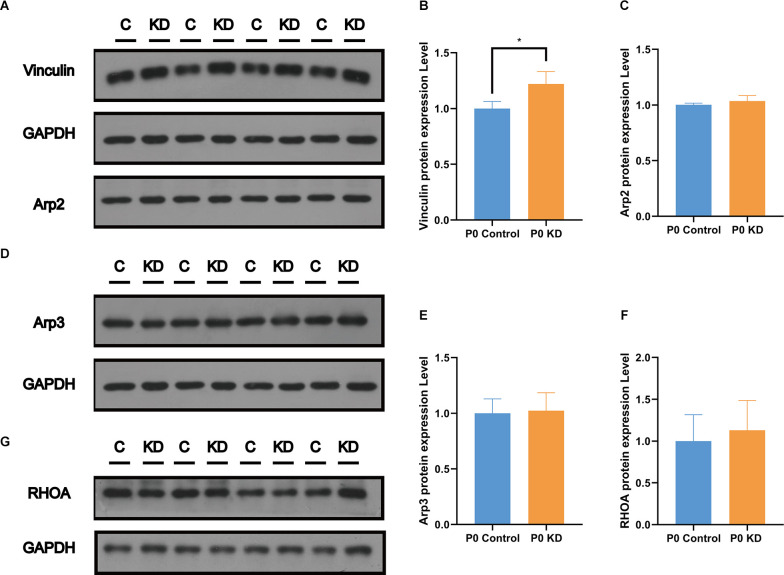
Significant increase of Vinculin was observed in the P0 KD group at P6. Protein expression level of Vinculin, Arp2 **(A)**, Arp3 **(D)**, and RHOA **(G)** in P0 control and P0 KD groups. Comparison of Vinculin **(B)**, Arp2 **(C)**, Arp3 **(E)**, and RHOA **(F)** by western blot in P0 control and P0 KD groups. The differences of Arp2, Arp3, and RHOA expression level between the P0 KD and the P0 control group were not significant (*P* > 0.05). *Significantly different from the control group (*P* < 0.05).

Cytoskeleton assembly is regulated in a complex manner within the cell. There are many actin-binding proteins which play important roles in maintaining actin cytoskeleton stability. Vinculin enables mechanical coupling between the actin cytoskeleton and the extracellular matrix (Kelley et al., 2020). Changes in the expression level of vinculin have been found to reshape actin filaments or regulate their assembly (Kelley et al., 2020). In order to investigate the mechanism of action of Cx26 KD on actin filaments, we analyzed the protein expression levels of vinculin, Arp2, Arp3, and RHOA. Compared with the control group, there were no significant differences in the protein expression levels of Arp2, Arp3, or RHOA (Figures 7A–F). However, compared with the control group, the content of vinculin in the P0 KD group increased by 22% (Figure 7A). This suggests that vinculin may be involved in the development of actin filaments in the OC caused by Cx26 KD.

### LRRK Decreased in PCs of the P0 KD Group

Previous studies have reported that LRRK2 regulates the microfilament cytoskeleton, and its loss causes a decrease in the F-actin content in neuronal filopodia (Caesar et al., 2015; Yan et al., 2020). One of the mechanisms by which LRRK2 regulates the actin cytoskeleton is its interaction with the small GTPases CDC42 and Rac1, both of which are key factors for regulating actin filaments. In order to investigate the mechanism underlying the effect of Cx26 KD on actin filaments, we analyzed the protein expression level of LRRK2. Compared with the control group, the fluorescence intensity of LRRK2 in the P0 KD group was reduced by 97%. This suggests that LRRK2 may be involved in the abnormal development of actin filaments in the OC caused by Cx26 KD.

## Discussion

### Deformity of the OC May Be an Important Reason for Congenital *Gjb2*-Related Hearing Loss

In previous studies, it was found that several strains of transgenic mouse models with reduced cochlear Cx26 suffered from serious hearing loss, accompanied by cochlear developmental disorders, loss of hair cells, decreased intracochlear potential, and the reduction of active cochlear amplification (Griffith et al., 2006; Zhao and Yu, 2006; Kibschull et al., 2008). Pathological phenomena of abnormal development, such as failure of the TC to open and disappearance of Nuel’s space have also been observed in some human cases (Wang et al., 2009). Circulatory disturbance of potassium ions was considered to be the main mechanism of deafness in Cx26-null mice. These studies and our previous study have shown that knockdown *Gjb2* in the early stage can cause severe hearing loss (Figures 1A,B) in mice and abnormal morphology of the OC (Figures 2A,B), while loss of Cx26 in late inner ear development (P8) does not cause deformity of the OC (Figures 2C,D) or hearing loss (Figures 1A,B; Chen et al., 2018b). Therefore, deformity of the OC may be a particularly important reason for congenital *Gjb2*-related hearing loss.

### Cytoskeleton Development Disorder Due to *Gjb2* KD Contributes to OC Deformity

**Figure 8:**
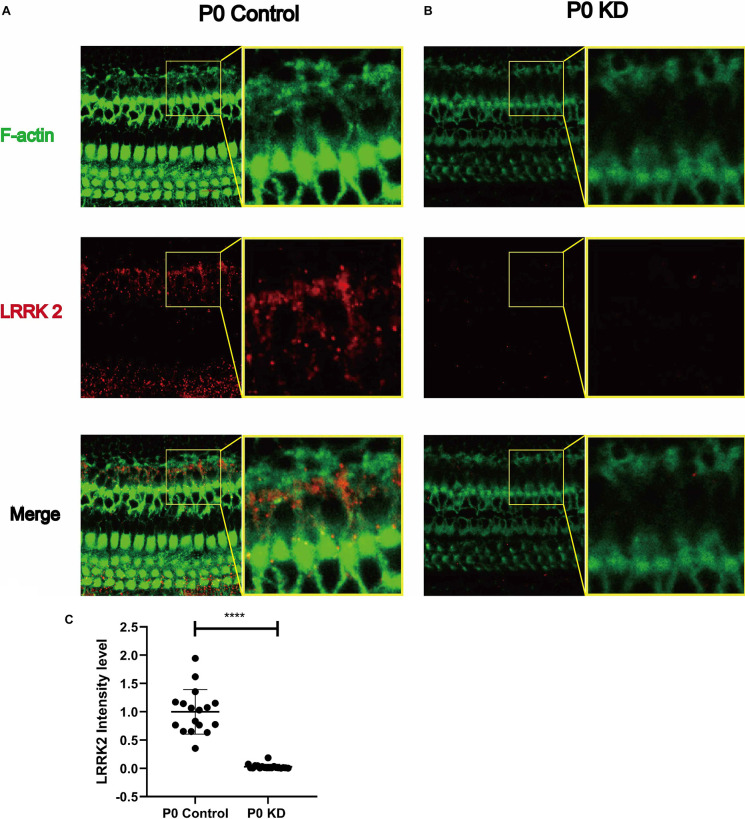
Significant reduction of LRRK2 was observed in the P0 KD group at P6. Immunofluorescence staining of LRRK2 in P0 control **(A)**, P0 KD groups **(B)**. Comparison of LRRK2 intensity between P0 KD group and P0 Control group **(C)**. ****Significantly different from the control group (*P* < 0.0001).

IPCs and OPCs together build the structure and shape of the TC. Our previous study suggested that deletion of the *Gjb2* gene in the early postnatal period causes microtubule abnormalities, which may be one of the mechanisms underlying the OC deformity of *Gjb2*-related hearing loss (Chen et al., 2018b). Actin filaments also play an important role in a variety of important life activities such as cell growth, deformation, and migration. In this experiment, we observed a significant decrease in the F-actin content of PCs and DCs in the P0 KD group at P6, while there was no significant difference in F-actin in the P8 KD group (Figures 3E–H,N,P, Figure 8). We tested the ratio of G-actin/F-actin, and the results showed that the ratio of G-actin/F-actin increased by 38% in the P0 KD group (Figure 5A). These results suggest that knockdown Cx26 in the early postnatal period will also cause abnormal development of actin filaments. The disordered cytoskeleton of PCs (both microtubule and microfilament) was essential for OC molding. Since OHCs do not express Cx26, Cx26 KD that occurs in PCs may not affect the actin cytoskeleton of hair cells.

### Reduction of LRRK2 Due to *Gjb2* KD May Be the Reason for Cytoskeleton Development Disorder

In order to identify the cause of the disordered microfilament network, we analyzed the mRNA and protein levels of G-actin (Figures 4A–E, 5D–I) and actin-binding proteins (4F–I, Figures 6, 7, 8) in the P0 KD group. The results showed that neither the mRNA level nor the protein level of β-actin and γ-actin has changed. In addition, the protein levels of the components of the cadherin complex connected to the microfilament network on the cell membrane, such as E-cadherin, α-catenin, β-catenin, and p120-catenin, were not changed (Griffith et al., 2006). Furthermore, we tried to detect some molecules that regulate F-actin network assembly. Our research showed that the Arp2/3 complex played an important role in F-actin assembly and nucleation (Figures 7A–F; Tang and Brieher, 2012). However, there were no significant changes in the protein level of Arp2 or Arp3.

We observed that vinculin increased by 22% in the P0 KD group. Vinculin is a cytoplasmic actin-binding protein, which directly binds to actin, stimulating actin polymerization and recruiting actin remodeling proteins (Bays and DeMali, 2017). Vinculin binds actin filaments to grow focal adhesions and regulates actin dynamics, and studies have shown that the tail domain of vinculin inhibits the extension of the F-actin barbed ends (Le Clainche et al., 2010). Vinculin stimulates the formation of new F-actin bundles in cells (Kovacs et al., 2011). Thus the increase of vinculin may be the cells’ response to abnormal skeletal development, to compensate by increasing the generation of new actin filaments.

We observed that LRRK2 in the P0 KD group was reduced by 97% (Figure 8). In previous studies of Parkinson’s disease, LRRK2 was found to be associated with changes in the cytoskeleton (Russo et al., 2014; Harvey and Outeiro, 2019). Under the stoichiometric conditions tested *in vitro*, Lrrk2 decreased the number of polymerized actin filaments, thus affecting the G-actin/F-actin ratio in favor of the monomer. Depletion of Lrrk2 in NIH3T3 cells leads to significant morphological alterations, which suggests that the cytoskeleton of NIH3T3 cells is affected (Meixner et al., 2011). In another study, up-regulation of LRRK2 caused by VIP was found to significantly reduce the F-/G-actin ratio (Niu et al., 2012). Thus the reduction of LRRK2 due to *Gjb2* KD may be the underlying reason for the cytoskeleton development disorder. A large part of LRRK2 research has involved the study of its interactions with small GTPases. The Rho-family of small GTPases is known as the master regulators of the actin cytoskeleton (Yan et al., 2020). The majority of previous studies were focused on RhoA, Rac1, and Cdc42. LRRK2 has been shown to bind RAC1, which increases the interaction of rac1 and p21-activated kinase and enhances rac1 activity, resulting in the regulation of actin cytoskeleton dynamics.

We observed reduced numbers of actin filaments in the malformed OC in Cx26 KD mice. We believe that the disordered development of the cytoskeleton composed of actin filaments and microtubules is the major reason for deformity of the OC.

## Data Availability Statement

The original contributions presented in the study are included in the article, further inquiries can be directed to the corresponding author/s.

## Ethics Statement

The animal study was reviewed and approved by Committee on Animal Research of Tongji Medical College, Huazhong University of Science and Technology.

## Author Contributions

YS and W-jK conceived and designed the study, reviewed and edited the manuscript. X-zL, YJ, SC, KX, LX, YQ, and X-hW performed the experiments. X-zL and YJ wrote the manuscript. All authors contributed to the article and approved the submitted version.

## Conflict of Interest

The authors declare that the research was conducted in the absence of any commercial or financial relationships that could be construed as a potential conflict of interest.

## Publisher’s Note

All claims expressed in this article are solely those of the authors and do not necessarily represent those of their affiliated organizations, or those of the publisher, the editors and the reviewers. Any product that may be evaluated in this article, or claim that may be made by its manufacturer, is not guaranteed or endorsed by the publisher.
